# Extensive Scalp Melanoma in an Elderly Female: A Case Report and Literature Review

**DOI:** 10.7759/cureus.45425

**Published:** 2023-09-17

**Authors:** Maya A Francis, Rahila Shaikh, Pugazhendi Inban, Ogbonnaya Akuma, Tarsha A Intsiful, Chinaza M Akuma, Lynn lat lat aung, Vivian C Chukwuedozie, Sandra Francis, Mikhail Sukhoroslov

**Affiliations:** 1 Internal Medicine, Windsor University School of Medicine, Cayon, KNA; 2 Dermatology/Internal Medicine, Saba University School of Medicine, The Bottom, BES; 3 General Medicine, Government Medical College, Omandurar, Chennai, IND; 4 Internal Medicine, Ebonyi State University, Abakaliki, NGA; 5 College of Medicine, University of Ghana Medical Center, Accra, GHA; 6 Public Health, Chamberlain University, Chicago, USA; 7 College of Medicine, MAHSA (Malaysian Allied Health Sciences Academy) University, Petaling Jaya, MYS; 8 Internal Medicine, S.M. Kirov Military Medical Academy, Saint Petersburg, RUS

**Keywords:** pathology, treatment, melanoma, cutaneous malignancy, elderly population, prognosis, diagnosis, scalp, pagetoid, nodular melanoma

## Abstract

Scalp melanoma is a rare and aggressive form of skin cancer. Its occurrence in the elderly population poses unique challenges due to factors such as delayed diagnosis and comorbidities. We present a case of extensive scalp melanoma in an elderly female to highlight the clinical presentation, diagnostic process, treatment modalities, and outcomes. Biopsy and histopathological analysis showed the presence of dysplastic nevi arising in pigmented melanocytic nevi, with uncertain pagetoid spread of atypical melanocytes. The management involved complete excision with safety margins and immunotherapy based on melanoma guidelines. This case underscores the importance of early detection and tailored treatment strategies in managing melanoma in elderly patients.

## Introduction

Cutaneous melanoma, the most fatal form of skin cancer, is known to be influenced by a combination of genetic and environmental factors [1]. While it can manifest in various parts of the body, cutaneous melanoma originating on the head accounts for 12-26%, with scalp melanomas representing approximately 5% of these cases [1,2]. Scalp melanomas are often referred to as the "invisible killers" due to their challenging location for self-examination, which can result in missed lesions [2]. Consequently, scalp melanomas tend to have a more unfavorable prognosis and a higher mortality rate than melanomas occurring in other anatomical regions of the body [3]. This report discusses the clinical presentation, histopathological findings, diagnostic challenges, and management of an extensive scalp melanoma found in an elderly female.

## Case presentation

**Figure 1:**
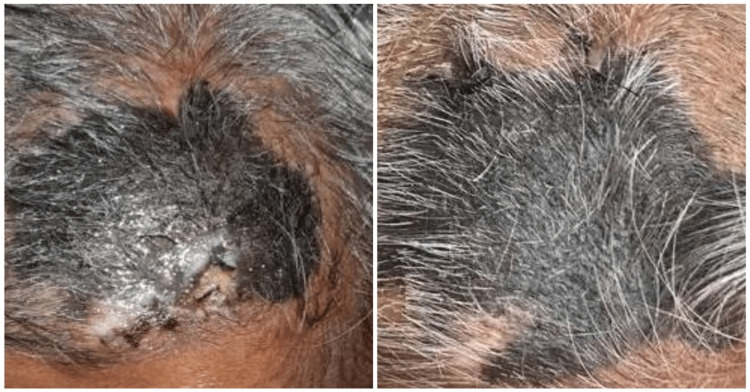
Scalp lesion upon initial presentation

A 64-year-old female presented to the dermatology outpatient clinic for evaluation of an extensive large, pigmented lesion on the scalp that had been present for the past several years and had progressively increased in size over the past year and a half. The patient had no significant past medical history or co-morbidities. The patient's family history did not include any skin or other cancers. The patient did not practice sun-protective behaviors such as the use of sunscreen or sun-protective clothing. The physical examination revealed an 8cm x 6cm dark-pigmented patch with scattered tufts of coarse and lustreless hair with no cervical and neck lymphadenopathy (Figure 1). The remaining systemic examination was unremarkable.

**Figure 2:**
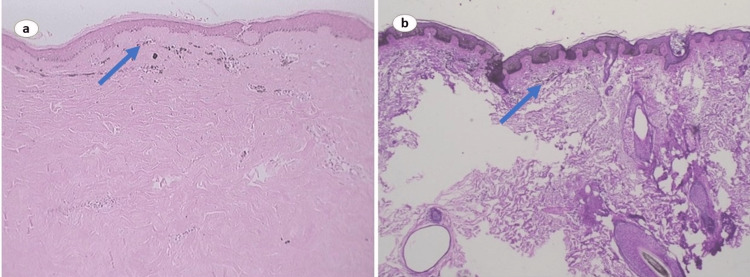
(a) Numerous melanocytes in the upper dermis; (b) Melanocytes appear loosely arranged in large groups in the upper dermis without well-circumscribed borders

On further assessment, two blocks and two hematoxylin-and-eosin-stained slides were obtained from a pigmented scalp lesion showing dysplastic nevi arising in a pigmented melanocytic nevus. However, the assessment of the pagetoid spread of atypical melanocytes remained uncertain. Cytological analysis exhibited several noteworthy features, including architectural alteration, fusion of papillae, focal atypia of melanocytes, delicate feathery cytoplasm, dermal fibrosis, and marked pigmentation. Further, an incisional biopsy of the edge of the lesion revealed melanocytic proliferation, with primarily displayed melanocyte proliferation confined to the lower epidermis and the dermo-epidermal junction. The melanocytes were arranged in solitary units, often occurring in pairs in close proximity. In some regions, the melanocytes nearly encompassed the entire dermo-epidermal junction and extended along the appendages. The edge of the lesion exhibited an indistinct boundary, with occasional melanocytes noted in the lower epidermis. Notably, no nests were observed at the junction or in the dermis. Additionally, numerous melanophages were present in the upper dermis, as illustrated in Figure 2a. These findings suggested the likelihood of the edge representing melanoma in situ; however, they were not definitive. Thus, obtaining a biopsy from the thickened center of the lesion was recommended to enable better classification and clinical correlation. 

Subsequently, another incisional biopsy demonstrated a melanocytic neoplasm involving the epidermis and dermis. Numerous melanocytes were observed at the dermo-epidermal junction and the lower epidermis, exhibiting a solitary pattern while closely arranged in a continuous run. Ill-formed melanocytic nests were occasionally seen, and a few larger, well-formed nests were present at the dermo-epidermal junction. Pagetoid melanocytes were identified above the basal layer and the stratum corneum in certain areas, accompanied by numerous melanin nuggets. The dermis had prominent collections of epithelioid melanocytes characterized by abundant cytoplasm, large nuclei, and abundant melanin.

Although no nests were detected in the dermis, the melanocytes appeared loosely arranged in large groups in the upper dermis. The neoplasm displayed irregular distribution without well-circumscribed borders, as depicted in Figure 2b. Based on the clinical features and the aforementioned findings, the impression strongly suggested nodular pagetoid melanoma with a thickness of 1mm (Clarke level 3). The patient underwent surgical excision of the lesion with 2-cm margins followed by BRAF inhibitor therapy.

The patient started to recover well on further follow-ups and the follow-up care plan included annual skin examinations with the dermatologist. Also, the patient was educated about signs and symptoms that required immediate follow-ups such as ulcerations, itching, bleeding, color variations, and increasing diameter of skin lesions. 

## Discussion

The histopathological features, combined with the clinical presentation, necessitated a management plan that involved complete excision of the melanocytic lesion with at least 1cm to 2cm safety margins. Additionally, sentinel lymph node dissection was recommended for staging the lesion, particularly with 1cm or more deep tumors. Following staging, an immunotherapy regimen may be considered in addition to the treatment regime due to the severity and potential aggressiveness of melanocytic lesions, following current cancer guidelines for melanoma [4].

This case report highlights the challenges of diagnosing atypical, pigmented scalp lesions. The presence of dysplastic nevi in pigmented melanocytic nevi and the questionable pagetoid spread of atypical melanocytes added complexity to the diagnostic process. The histopathological findings, including the absence of nests in the dermis and the presence of pagetoid melanocytes, were consistent with diagnosing pagetoid melanoma nodular type. The nodular subtype of melanoma accounts for more than 40% of melanoma deaths compared to other types and may mimic benign skin lesions [5].

Diagnosing melanocytic lesions remains a persistent challenge in medicine due to their potential for rapid metastasis and associated mortality, which are closely linked to tumor thickness and invasion [6]. Late detection is associated with increased thickness and invasion, highlighting early detection's importance. Also, the wide spectrum of melanocytic morphology presents a challenge in medicine, as it encompasses various subtypes such as nodular, acral lentiginous, and superficial spreading, further complicating the diagnostic process [6]. As our understanding of melanocytic lesions continues to evolve, new subtypes are continually being recognized, adding to the complexity of accurate diagnosis and classification. Given the implications for patient outcomes, enhancing our diagnostic capabilities and ensuring timely detection of melanocytic lesions is crucial to optimize management strategies and improve overall prognosis.

Therefore, regular skin examinations are important in early skin lesion detection, particularly in hard-to-observe areas like the scalp. Timely identification of such lesions through skin examinations has significantly reduced mortality rates [7]. Encouraging patients to actively engage in self-skin examinations is a valuable secondary health prevention method and empowers them to take charge of their healthcare [7,8]. In this context, educational tools like the melanoma ABCDE (asymmetry, border, color, diameter and evolving) rule are pivotal in aiding patients to recall skin cancer detection techniques [7,8]. Patients should also be educated on preventative measures such as daily use of sunscreen, sun protective clothing, and avoiding excess sun exposure. However, there remain notable gaps in approaching, adhering to, and conducting comprehensive skin examinations for patients. Physicians must identify and address these gaps when educating patients and highlighting the significance of regular self-skin examinations during medical visits, especially in high-risk individuals [9]. By doing so, earlier detection can be facilitated, improving patient prognoses.

Further studies and larger case series are warranted to understand better the clinical and pathological characteristics of scalp Pagetoid melanoma nodular type and to guide optimal management strategies for patients presenting with similar lesions [10]. This case is a valuable opportunity to raise awareness about atypical melanoma scalp lesions and educate patients. By sharing and discussing this specific case, we aim to increase knowledge and understanding of this particular aspect of nodular melanoma. Through increased awareness, healthcare professionals and individuals can be better equipped to recognize and diagnose atypical melanoma lesions on the scalp, leading to earlier detection and improved outcomes.

## Conclusions

This case report highlights the significance of recognizing and managing scalp melanoma in elderly patients. Timely diagnosis, appropriate surgical intervention, and tailored treatment strategies are essential in improving outcomes for this high-risk population. Moreover, this case highlights the critical role of regular skin examinations, particularly for hard-to-observe areas like the scalp, in facilitating the early detection of skin lesions. Identifying such lesions timely, encouraging patients to engage in self-skin examinations actively, and educating them on skin cancer detection techniques may reduce mortality rates. Further research is needed to better understand the optimal management of extensive scalp melanoma in elderly individuals and to develop guidelines that consider their specific needs and challenges.
